# A Cross-sectional Retrospective Analysis of Glycemic Burden and Nephropathy in an Indian Population and Formulation of a New Plan Using eGFR/HbA1c Grid Formation

**DOI:** 10.7759/cureus.5378

**Published:** 2019-08-13

**Authors:** Sayak Roy, Kingshuk Bhattacharjee

**Affiliations:** 1 Internal Medicine, Calcutta Medical Research Institute Hospital, Kolkata, IND; 2 Medicine, Shri Jagdishprasad Jhabarmal Tibrewala University, Jhunjhunu, IND

**Keywords:** glycated hemoglobin, estimated glomerular filtration rate, type 2 diabetes, nephropathy, low-middle income country

## Abstract

Background

Diabetes is a metabolic, non-communicable disease (NCD) that represents one of the major causes of morbidity and mortality worldwide. India has a huge burden of chronic kidney disease (CKD) that is associated with diabetes.

Materials and methods

Cross-sectional data were collected for a total of 241 patients from the authors’ clinic record. A new approach for the management of patients with diabetes was proposed using a grid system, where we need to consider the Hemoglobin A1C (HbA1c) and estimated glomerular filtration rate (eGFR) values of the patient and assign a zone (green, blue, orange or red) and subsequently decide an appropriate treatment according to the assigned zone.

Results

We found that 20.73% of patients had decreased eGFR and only 31.12% of patients achieved target HbA1c level. A high prevalence of diabetic nephropathy (20.73%) was observed in this study population. A statistically significant difference among the four groups (zones) with respect to age (*p* <0.001), duration of diabetes (*p* = 0.024), HbA1c (*p* <0.001), and eGFR (*p* <0.001) was found.

Conclusion

The burden of diabetes and nephropathy is high in low-income countries and can be easily assessed by applying simple tools such as the newly proposed HbA1c/eGFR grid system to identify high-risk and medium-risk patients and adopting treatment according to the assigned zone.

## Introduction

Diabetes is a metabolic, non-communicable disease (NCD) that is one of the leading causes of morbidity and mortality worldwide. After emerging as a concern in developed countries, diabetes has now become a major healthcare concern in developing countries, especially in urban areas, owing to the sedentary lifestyle. In India, considerable success has been achieved in controlling communicable and infectious diseases, but NCDs (like diabetes) are becoming more common currently and represent a significant burden on healthcare due to concomitant disorders [1-2]. According to the World Health Organization (WHO), the NCDs were the cause of about 60% of all the deaths in India, in 2012. The NCDs included cardiovascular diseases (26%), chronic respiratory diseases (13%), cancer (7%), diabetes (2%), and other NCDs (12%) [3-4]. The International Diabetes Federation (IDF) has estimated the prevalence of diabetes to be 8.8% in India, in 2017 [5].

When it comes to the treatment for diabetes in a low to medium socio-economic zone, several drug molecules, although successfully validated by cardiovascular outcome trials (CVOT), cannot be used, particularly by the underprivileged patient population, owing to their exorbitant prices. Hence, age-old drugs such as pioglitazone and sulphonylureas (SU) are considered for glycated hemoglobin (HbA1c) control. In India, several molecules have been approved for the treatment of diabetes, including teneligliptin, saroglitazar, and the latest remogliflozin, which are cheaper than their CVOT holding counterparts.

India is having a huge burden of chronic kidney disease (CKD) and diabetes is among the major contributing factors in the development of CKD. Due to lack of access to healthcare in India, more than 50% of patients with CKD receive an intervention when the eGFR is already below 15 mL/min per 1.73 m^2^ [6]. The high burden of HbA1c and nephropathy are interlinked and both form the common soil of CVD in the Indian population. Hence, to tackle this huge burden, a new treatment plan is necessary that can be easily interpreted by the medical practitioners who are the first point of contact for the diabetic population and who deliver the treatments. The international guidelines are often too cumbersome to remember and practice, and thus, a new easy-to-use tool can help the medical practitioners to achieve the target of good glycemic control along with the management of nephropathy. Here we propose for the first time a new model that illustrates our action plan based on the prevailing HbA1c and estimated glomerular filtration rate (eGFR) of the patient.

Hypothesis

This new approach uses of a grid system where we need to consider the HbA1c and eGFR values of the patient and assign a zone in which the patient is lying and subsequently decide an appropriate treatment for the patient. There are 4 different color-coded zones proposed for this purpose.

Significance of each zone

Green Zone

Green zone includes patients with normal eGFR and normal HbA1c level, and hence, we need to continue the same as the patient was taking before if there is no comorbid condition and/or complication which needs to be addressed. However, we might, in certain cases, reduce the HbA1c further if there is no unwanted side-effect.

Blue Zone

Blue zone includes patients with normal eGFR but increased HbA1c level, and hence, the action plan will be controlling the HbA1c level only without much consideration about the kidneys (provided that there is no microalbuminuria that may lead to the development of microvascular complications if not controlled properly).

Orange Zone

Orange zone includes patients with decreased eGFR but normal HbA1c. These patients require special attention for possible restoration of their kidney function or halting further deterioration of their current kidney status because some drugs like sodium-glucose cotransporter 2 inhibitor (SGLT2I) can cause a physiological drop in the eGFR.

Red Zone

Red zone includes patients with high HbA1c and low eGFR. This group needs special care with multiple drugs that can be given in controlled doses and this group of patients might require a frequent follow-up.

## Materials and methods

Cross-sectional data were collected for a total of 241 patients from the authors’ clinic record for the purpose of this analysis. Reduced eGFR was defined by a value less than 60 ml/ min/ 1.73 m^2^, and increased HbA1c was defined by a value of more than 7%. The study was done in accordance with the principles laid down in the Declaration of Helsinki. Informed consent was obtained from all the participants. Patients were divided into four zones (or four cohorts: green, blue, orange, and red zone cohorts) based on their baseline HbA1c levels and eGFR, and data from these patients were analyzed in context with the objectives of this study.

For the comparison of results obtained from each zone, analysis of variance (ANOVA) test was used for the calculation of *p*-value, which included various dependent variables (age, duration of diabetes, HbA1c, and eGFR) in each of the four groups. Dunnett T3 multiplicity test was applied to assess the reason for statistical significance (if any) found by ANOVA.

Objectives of the analysis

Patients were divided into four zones (or four cohorts: green, blue, orange, and red zone cohorts) based on their baseline HbA1c levels and eGFR and data from these patients were analyzed in context with the objectives of this study. The primary outcomes for analysis were the burden of glycemic status and nephropathy at baseline over a cross-sectional timeline, percentage of patients in each zone at baseline, mean value of HbA1c in each cohort, mean eGFR in each cohort, mean age of each cohort, and mean duration of diabetes in each cohort.

Secondary outcomes for analysis were percentage of patients with insulin use in the said population over a fixed cross-section of time, percentage of patients with SGLT2I use, percentage of patients with SU use, correlation of baseline HbA1c with duration of diabetes, correlation of baseline eGFR with duration of diabetes and percentage of patients with the use of teneligliptin.

## Results

It was found that 31.12% (n = 75) of patients were in the green zone, 48.13% (n =116) were in the blue zone, 10.78% (n = 26) were in orange zone, and 9.95% (n = 24) of patients were in the red zone. In this cross-sectional data analysis, we found that 20.73% of patients had decreased eGFR and only 31.12% of patients achieved target HbA1c level. On applying ANOVA test, it was found that there was a statistically significant difference among the four groups with respect to age (*p* <0.001; Table 1), duration of diabetes (*p* = 0.024; Table 2), HbA1c (*p* <0.001; Table 3), and eGFR (*p* <0.001; Table 4).

**Table 1:** Results obtained using ANOVA test with “age” as a dependent variable P-value of <0.05 indicated a statistically significant difference among the four groups; SD = standard deviation; ANOVA = analysis of variance

Descriptive Statistics							
Baseline zone		N	Minimum	Maximum	Mean	SD	P-Value (ANOVA)
Green zone cohort	Age	76	34	84	57.84	10.957	<0.001
Orange zone cohort	Age	24	52	78	65.63	6.309	
Blue zone cohort	Age	117	19	86	53.80	12.293	
Red zone cohort	Age	24	48	80	64.13	8.142	

**Table 2:** Results obtained using ANOVA test with “duration of diabetes” as a dependent variable *P*-value of <0.05 indicated a statistically significant difference among the four groups; D/DIAB = duration of diabetes; SD = standard deviation; ANOVA = analysis of variance

Descriptive Statistics							
Baseline zone		N	Minimum	Maximum	Mean	SD	P-value (ANOVA)
Green zone cohort	D/DIAB	76	0.20	20.00	6.706	4.308	0.024
Orange zone cohort	D/DIAB	24	1.00	18.00	8.958	5.212	
Blue zone cohort	D/DIAB	117	0.01	20.00	7.584	5.047	
Red zone Cohort	D/DIAB	24	0.11	20.00	9.879	5.415	

**Table 3:** Results obtained using ANOVA test with “HbA1c” as a dependent variable P-value of <0.05 indicated a statistically significant difference among the four groups; HbA1c = glycated hemoglobin; SD = standard deviation; ANOVA = analysis of variance

Descriptive Statistics							
Baseline ZONE		N	Minimum	Maximum	Mean	SD	P-Value (ANOVA)
Green zone Cohort	HbA1c	76	5	7	6.36	0.626	< 0.001
Orange zone Cohort	HbA1c	24	5	8	6.42	0.654	
Blue zone Cohort	HbA1c	117	6	16	8.78	1.801	
Red zone Cohort	HbA1c	24	7	14	9.83	2.278	

**Table 4:** Results obtained using ANOVA test with “eGFR” as a dependent variable *P*-value of <0.05 indicated a statistically significant difference among the four groups; eGFR = estimated glomerular filtration rate; SD = standard deviation; ANOVA = analysis of variance

Descriptive Statistics							
Baseline zone		N	Minimum	Maximum	Mean	SD	P (ANOVA)
Green zone cohort	eGFR	76	60	121	82.55	14.164	<0.001
Orange zone cohort	eGFR	24	33	59	48.58	7.604	
Blue zone cohort	eGFR	117	61	136	88.64	16.581	
Red zone Cohort	eGFR	24	18	59	45.25	11.448	

To determine the reason for the statistically significant difference observed in ANOVA analysis, multiplicity comparison among the groups was done using the Dunnett T3 test with age (Table 5), duration of diabetes (Table 6), HbA1c (Table 7), and eGFR (Table 8) as dependent variables.

**Table 5:** . Multiplicity comparisons using the Dunnett T3 test with “age” as a dependent variable *Statistically significant difference between the two groups at the 0.05 level; SE = standard error

(I)	(J)	Mean Difference (I-J)	SE	P-value	95% Confidence Interval	
					Lower Bound	Upper Bound
Green zone cohort	Orange zone cohort	-7.783^*^	1.800	<0.001	-12.65	-2.91
	Blue zone cohort	4.039	1.694	0.104	-0.47	8.55
	Red zone cohort	-6.283^*^	2.084	0.023	-11.97	-0.59
Orange zone Cohort	Green zone cohort	7.783^*^	1.800	<0.001	2.91	12.65
	Blue zone cohort	11.822^*^	1.718	<0.001	7.17	16.48
	Red zone cohort	1.500	2.103	0.978	-4.28	7.28
Blue zone cohort	Green zone cohort	-4.039	1.694	0.104	-8.55	0.47
	Orange zone cohort	-11.822^*^	1.718	<0.001	-16.48	-7.17
	Red zone cohort	-10.322^*^	2.013	<0.001	-15.84	-4.81
Red zone cohort	Green zone cohort	6.283^*^	2.084	0.023	0.59	11.97
	Orange zone cohort	-1.500	2.103	0.978	-7.28	4.28
	Blue zone cohort	10.322^*^	2.013	<0.001	4.81	15.8

**Table 6:** Multiplicity comparisons using the Dunnett T3 test with “duration of diabetes” as a dependent variable *Statistically significant difference between the two groups at the 0.05 level; SE = standard error

(I)	(J)	Mean Difference (I-J)	SE	P-value	95% Confidence Interval	
					Lower Bound	Upper Bound
Green zone cohort	Orange zone cohort	-2.25175	1.17319	0.313	-5.5202	1.0167
	Blue zone cohort	-0.87744	.67968	0.731	-2.6850	0.9301
	Red zone cohort	-3.17300^*^	1.21081	0.045	-6.5510	0.2050
Orange zone cohort	Green zone cohort	2.25175	1.17319	0.313	-1.0167	5.5202
	Blue zone cohort	1.37432	1.16187	0.799	-1.8686	4.6173
	Red zone cohort	-0.92125	1.53428	0.991	-5.1296	3.2871
Blue zone cohort	Green zone cohort	.87744	0.67968	0.731	-0.9301	2.6850
	Orange zone cohort	-1.37432	1.16187	0.799	-4.6173	1.8686
	Red zone cohort	-2.29557	1.19984	0.318	-5.6491	1.0580
Red zone cohort	Green zone cohort	3.17300^*^	1.21081	0.045	-0.2050	6.5510
	Orange zone cohort	0.92125	1.53428	0.991	-3.2871	5.1296
	Blue zone cohort	2.29557	1.19984	.318	-1.0580	5.6491

**Table 7:** Multiplicity comparisons using the Dunnett T3 test with “HbA1c” as a dependent variable * Statistically significant difference between the two groups at the 0.05 level; HbA1c = glycated hemoglobin; SE = standard error

(I)	(J)	Mean Difference (I-J)	SE	P-value	95% Confidence Interval	
					Lower Bound	Upper Bound
Green zone cohort	Orange zone cohort	-0.061	.152	0.999	-0.48	0.36
	Blue zone cohort	-2.423^*^	.181	<0.001	-2.91	-1.94
	Red zone cohort	-3.478^*^	0.470	<0.001	-4.82	-2.14
Orange zone Cohort	Green zone cohort	0.061	0.152	0.999	-0.36	0.48
	Blue zone cohort	-2.361^*^	0.213	<0.001	-2.93	-1.79
	Red zone cohort	-3.417^*^	0.484	<0.001	-4.78	-2.05
Blue zone Cohort	Green zone cohort	2.423^*^	0.181	<0.001	1.94	2.91
	Orange zone cohort	2.361^*^	0.213	<0.001	1.79	2.93
	Red zone cohort	-1.056	0.494	0.213	-2.44	0.33
Red zone Cohort	Green zone cohort	3.478^*^	0.470	<0.001	2.14	4.82
	Orange zone cohort	3.417^*^	0.484	<0.001	2.05	4.78
	Blue zone cohort	1.056	0.494	0.213	-0.33	2.44

**Table 8:** Multiplicity comparisons using the Dunnett T3 test with “eGFR” as a dependent variable *Statistically significant difference between the two groups at the 0.05 level; eGFR = estimated glomerular filtration rate; SE = standard error

(I)	(J)	Mean Difference (I-J)	SE	P-value	95% Confidence Interval	
					Lower Bound	Upper Bound
Green zone cohort	Orange zone cohort	33.969^*^	2.247	<0.001	27.90	40.04
	Blue zone cohort	-6.088^*^	2.234	0.041	-12.03	-0.15
	Red zone cohort	37.303^*^	2.846	<0.001	29.50	45.10
Orange zone cohort	Green zone cohort	-33.969^*^	2.247	<0.001	-40.04	-27.90
	Blue zone cohort	-40.058^*^	2.182	<0.001	-45.94	-34.17
	Red zone cohort	3.333	2.805	0.796	-4.41	11.08
Blue zone cohort	Green zone cohort	6.088^*^	2.234	0.041	0.15	12.03
	Orange zone cohort	40.058^*^	2.182	<0.001	34.17	45.94
	Red zone cohort	43.391^*^	2.795	<0.001	35.72	51.06
Red zone cohort	Green zone cohort	-37.303^*^	2.846	<0.001	-45.10	-29.50
	Orange zone cohort	-3.333	2.805	.796	-11.08	4.41
	Blue zone cohort	-43.391^*^	2.795	<0.001	-51.06	-35.72

The Dunnett T3 test with “age” as dependent variable showed statistically significant difference for comparisons between the green zone and orange zone (*p* <0.001); green zone and red zone (*p* = 0.023); orange zone and blue zone (*p* <0.001); and between blue zone and red zone (*p* <0.001). When “duration of diabetes” was included as the dependent variable, the statistically significant difference was obtained for the comparison between the green zone and red zone (*p* = 0.045) only. When “HbA1c” was included as the dependent variable, a statistically significant difference was obtained for the comparisons between the green zone and blue zone (*p* <0.001); red zone and orange zone (*p* <0.0001); red zone and orange zone (*p* <0.001); and between blue zone and orange zone cohort (*p* <0.001). When “eGFR” was included as the dependent variable, a statistically significant difference (*p* <0.001) was obtained for all comparisons among the four zones except for the comparison between the red zone and orange zone.

A regression analysis was also performed to assess the correlation between the cross-sectional values for HbA1c and duration of diabetes (Table 9) and for eGFR and duration of diabetes (Table 10).

**Table 9:** Correlations between HbA1c and duration of diabetes over the cross-sectional period D/DIAB = duration of diabetes; HbA1C = glycated hemoglobin

Baseline Zone			D/DIAB
Green zone cohort	Baseline HbA1C	Pearson Correlation	0.042
		P-value (2-tailed)	0.721
		N	76
Orange zone cohort	Baseline HbA1C	Pearson Correlation	-0.365
		P-value (2-tailed)	0.080
		N	24
Blue zone cohort	Baseline HbA1C	Pearson Correlation	-0.257^**^
		P-value (2-tailed)	0.005
		N	117
Red zone cohort	Baseline HbA1C	Pearson Correlation	0.221
		P-value (2-tailed)	0.300
		N	24

**Table 10:** Correlations between eGFR and duration of diabetes over the cross-sectional period eGFR = estimated glomerular filtration rate; D/DIAB = duration of diabetes

Baseline Zone			D/DIAB
Green zone cohort	eGFR	Pearson Correlation	-0.250^*^
		P-value (2-tailed)	0.029
		N	76
	D/DIAB	Pearson Correlation	1
		P-value (2-tailed)	
		N	76
Orange zone cohort	eGFR	Pearson Correlation	-0.060
		P-value (2-tailed)	0.782
		N	24
	D/DIAB	Pearson Correlation	1
		P-value (2-tailed)	
		N	24
Blue zone cohort	eGFR	Pearson Correlation	-0.338^**^
		P-value (2-tailed)	0.000
		N	117
	D/DIAB	Pearson Correlation	1
		P-value (2-tailed)	
		N	117
Red zone cohort	eGFR	Pearson Correlation	-0.301
		P-value (2-tailed)	0.153
		N	24
	D/DIAB	Pearson Correlation	1
		P-value (2-tailed)	
		N	24

The correlation analysis for HbA1c and duration of diabetes indicated a statistical significance for the blue zone, suggesting a decrease in HbA1c with an increase in the duration of diabetes that might be a result of drug usage in this group. The regression analysis applied to assess the correlation between cross-sectional values of eGFR and duration of diabetes revealed statistical significance for the green zone (*p* = 0.029) and blue zone (*p* = 0.000) reflecting the fact that, in these two groups, eGFR decreased with the increase in the duration of diabetes (indicating the normal eGFR decline pattern with time).

A high prevalence of diabetic nephropathy (20.73%) was observed in this study population. Considering that all the eGFR values were second-time values noted after obtaining a reduced value three months before assessment indicate the severity of nephropathy observed in this study. Only 31.12% of patients achieved the target of 7% HbA1c level. High usage of SU group of drugs (*n* = 114; 47.3% of patients) and dipeptidyl peptidase-4 inhibitors (DPP4I) group of drugs (*n* = 112; 46.47% of patients) was observed in this study population. However, the use of costly SGLT2Is was seen in only 24.89% of patients (*n* = 60). This higher use of cheaper medicines for glycemic control as compared with the expensive molecules reflects the need for cheaper medicines in the middle-income countries where most patients have to pay the treatment cost from their own pockets and only a small number of patients are covered under insurance.

## Discussion

A systematic review of the literature containing data from the lower and middle-income countries (LMIC) showed the inadequacy of most LMIC guidelines in terms of applicability, clarity, and dissemination. In addition, most of the LMIC guidelines lack the socio-economic and ethical-legal contextualization as they mainly target healthcare providers, with only a few guidelines targeting patients (7%), expense payers (11%), and policymakers (18%) were found. The spectrum of diabetes mellitus (DM) clinical care addressed by LMIC guidelines was narrower compared with the high-income country guidelines [7]. Thus, there is an immense need for the development of a robust and easy-to-understand guideline for the treatment of the population with diabetes in LMIC.

In 2012, the Indian government’s healthcare expenditure was lower compared with other countries in the SEA region, with a general expense on healthcare representing 33% of total healthcare expenditure in India compared with an average expenditure of 52% in SEA countries [8]. In India, the healthcare expenditure is mainly borne by the patients with the government contributing to merely one-third of total healthcare expenditure of the country. The out-of-pocket expenses represent about 58% of total healthcare expenditure in 2012 [8]. In the Chennai Urban Rural Epidemiology Study (CURES 45), it was observed that the prevalence of diabetic nephropathy and microalbuminuria was 2.2 and 26.9%, respectively, in urban Asian-Indian population with type-2 diabetes. The most common risk factors identified for diabetes-associated diabetic nephropathy and microalbuminuria included the duration of diabetes, HbA1c, and systolic blood pressure [9].

A marked heterogenicity exists in the prevalence of diabetes throughout India. It has been reported that the urban population has a high prevalence of diabetes compared with the rural population. Also, different reports have suggested that the North Indian population (Chandigarh 0.12 million, Jharkhand 0.96 million) has lower diabetes prevalence compared with the Central (Maharashtra 9.2 million) and South Indian populations (Tamil Nadu 4.8 million). A similar trend was observed in a national survey that analyzed the prevalence of diabetes in metropolitan cities across India, indicating high prevalence in South India compared with other regions: East India: Kolkata (11.7%); North India: Kashmir Valley (6.1%) and New Delhi (11.6%); West India: Mumbai (9.3%) compared with South India: Chennai (13.5%), Hyderabad (16.6%), and Bangalore (12.4%) [10-12].

Poor glycemic control has been reported by different studies in Indian population. A hospital-based observational study carried out on 622 newly diagnosed subjects with type-2 DM who received treatment from August 2006 to January 2009 showed a mean HbA1c level of 9.02% ± 1.67. The outcome of good glycemic control (HbA1c level of <7%) was accomplished by 7.4% of patients. In patients who achieved good glycemic control, 41% had baseline HbA1c >9%, 26% had baseline HbA1c between 8% and 9%, and 26% had baseline HbA1c between 7% and 8% [13]. Poor glycemic control may lead to several complications related to the heart, kidneys, eyes, and brain. The complications related to kidney function are most commonly observed in Indian population and should be managed along with the glycemic control. This study highlights the need for the development of a new model to tackle the problem of diabetes and nephropathy simultaneously. The Diabetes Control and Complications Trial showed a statistically significant correlation between HbA1c levels and the risk of microvascular complications including CKD [14]. An observational cross-sectional study that involved patients with type-2 DM who were receiving concurrent treatment for diabetes and hypertension showed that the mean HbA1c level was significantly higher and eGFR was significantly lower in patients with baseline serum creatinine ≥1.2 mg/dL compared with patients with baseline serum creatinine <1.2 mg/dL. The results indicated that poor glycemic control leads to diabetic nephropathy [15]. The results are consistent with the data collected in the current study that indicated a high prevalence of diabetic nephropathy in the Indian population (20.73%).

Also, it was observed that a higher proportion of the study population used cheaper medications compared with the western population who generally use costly medications. Interestingly, the use of cheaper medicines and less use of costly molecules did not alter the epidemiological prevalence of uncontrolled glycemic burden when compared with developed western countries like the United States, where it has been reported that about 37% of patients with diabetes achieved the target level of <7% [16]. This cross-sectional study revealed the loopholes that remain in our current treatment plan and highlight the areas where the physicians need to be more careful. Proper utilization of the newly proposed grid model based on easily accessible parameters (HbA1c/eGFR) can be ideal for the treatment and follow-up of low-income patients.

## Conclusions

India is a resource-constraint country where most patients have to incur the cost of treatment by themselves. Thus, the 2018 American Diabetes Association and the European Association for the Study of Diabetes (ADA/EASD) joint consensus has aptly set a “price factor” in selecting an appropriate antidiabetic treatment. We can decrease the burden of diabetes and nephropathy using easy assessment tools such as the above-mentioned HbA1c/eGFR grid system to identify high-risk and medium-risk patients and then properly lay down an action plan. This case series involved a small number of patients. The clinical significance of the newly proposed model is required to be validated by recruiting a larger number of patients and then following them up for a longer duration while treating them according to the proposed model.
